# Dark Current Reduction and Performance Improvements in Graphene/Silicon Heterojunction Photodetectors Obtained Using a Non-Stoichiometric HfO_x_ Thin Oxide Layer

**DOI:** 10.3390/nano14050419

**Published:** 2024-02-25

**Authors:** Tao Qu, Jibin Fan, Xing Wei

**Affiliations:** School of Materials Science and Engineering, Chang’an University, Xi’an 710061, China; 2021131053@chd.edu.cn (T.Q.); weixing@chd.ed.cn (X.W.)

**Keywords:** magnetron sputtering, hafnium oxide, graphene photodetector, interfacial layer

## Abstract

Graphene/silicon heterojunction photodetectors suffer from a high dark current due to the high surface states and low barrier height at the interface, which limits their application. In this study, we introduce an HfO_x_ interfacial layer via magnetron sputtering to address this issue. With this new structure, the dark current is reduced by six times under a bias voltage of −2 V. Under 460 nm illumination, the responsivity is 0.228A/W, the detectivity is 1.15 × 10^11^ cmHz^1/2^W^−1^, and the noise equivalent power is 8.75 × 10^−5^ pW/Hz^1/2^, demonstrating an excellent weak light detection capability. Additionally, the oxygen vacancies in the HfO_x_ interfacial layer provide a conductive channel for charge carriers, resulting in a 2.03-fold increase in photocurrent and an external quantum efficiency of 76.5%. The photodetector maintains good photoresponse ability at a low bias voltage. This work showcases the outstanding performance of HfO_x_ films as interfacial layer materials and provides a new solution for high-performance photodetectors, as well as a new path to improve the photovoltaic conversion efficiency of solar cells.

## 1. Introduction

Graphene’s high mobility, atomic thinness, and zero bandgap make it an idea photosensitive material for photodetectors [1,2]. Generally, the spectral range of the photodetectors, such as GaN [3], Si [4], Ge [5], HgCdTe [6] and InGaAs [7], is determined by the bandgap of the semiconductors serving as the photosensitive material. Owing to the zero bandgap of graphene, graphene-based photodetectors have the ability to detect light signals from ultraviolet to far-infrared at room temperature [8]. Ultrabroadband responses at room temperature to graphene-based photodetectors have broad applications and they can also reduce the device’s cost. The zero-band structure of graphene led to ultrabroadband photodetection for graphene-based photodetectors, but it also generated a considerable dark current due to electron-hole generation. The photodetection in graphene-based photodetectors with a large dark current will be limited, especially for weak signals [9].

To solve this problem, many theoretical and experimental studies have been carried out. It was found that growing a thin insulate layer between graphene and silicon can increase the effective potential barrier height and significantly reduce the dark current. Li et al. [8] inserted a thin oxidized silicon dioxide between graphene and silicon; the dark current of the graphene/Si heterojunction photodetectors is reduced by an order of magnitude at zero bias voltage. Higher dielectric constants materials, such as HfO_2_ and Al_2_O_3_, which have a better leakage current suppression in MOSFET, are also demonstrated. Furthermore, Xu et al.’s [10] study indicates that the thickness increase in aluminum oxide as the interfacial layer between graphene/Si obtained through using an atomic layer deposition (ALD) can obtain a smaller dark current, but this hampers the photoresponsivity. Song’s research also found that with the increase in interface oxide thickness, the photocurrent of graphene-based silicon heterojunction decreases, and the light response decreases at low bias voltages due to the tunneling mechanism. The thickness of the oxide layer should be less than 1.5 nm to reconcile the contradiction between dark current and light response in graphene/Si photodetectors [11]. However, it is usually difficult to obtain good characteristics for such a thin film using ALD as the initial ALD reaction cycles cannot be well controlled. Meanwhile, a thin interfacial layer is commonly generated between the films deposited by ALD and the substate, which will increase the total thickness of the layer [12].

A non-chemical stoichiometric ratio of HfO_x_ film was used in Resistive random-access memory (RRAM), which established an initial leakage current in HfO_x_ [13]. However, the potential application of HfO_x_ in photodetectors has not been investigated at present. Therefore, in this paper, to decrease the dark current without deteriorating the light response, the potential application of the non-chemical stoichiometric ratio of HfO_x_ films is investigated in graphene/Si heterojunction photodetectors. 

## 2. Materials and Methods

**Figure 1:**
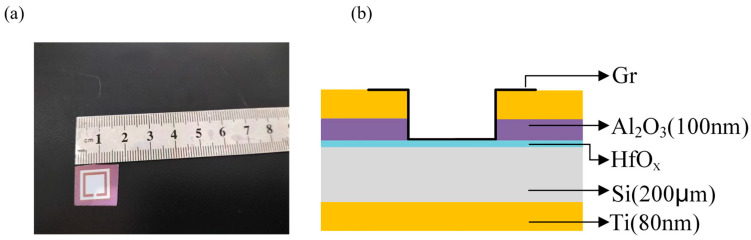
(**a**) Image of a graphene-based silicon Schottky photodetector with a HfO_x_ interfacial layer; (**b**) device structure diagram.

The physical diagram of the graphene photodetector is shown in Figure 1a. The device preparation process first involves cleaning n-type (100) silicon wafers with a resistivity of 2–4 Ω·cm using RCA, and then removing the natural oxide layer on the silicon wafer surface with a diluted HF solution (1:50). Subsequently, a non-stoichiometric HfO_x_ thin film was grown using an RF magnetron sputtering system and high-purity hafnium oxide target (99.99%). The growth conditions included a sputtering power of 70 W, argon flow rate of 30sccm, and pre-sputtering for 2 min, followed by formal sputtering of HfO_x_ thin films of different thicknesses and durations. During the sputtering process, the reflection power was 18 W. The insulating layer (100 nm) of alumina (99.99%) and titanium (99.99%) electrodes (80 nm) were prepared using a pattern mask (1.1 cm × 1.1 cm). The photodetector, as shown in Figure 1b, had a vertical structure. The graphene of the photodetector was grown in a graphene chemical vapor deposition system (G-CVD), using methane as the carbon source and hydrogen as the cleaning and reducing agent, causing methane to crack at 1030 °C in a high vacuum, depositing carbon on copper foil (alfa aesar, 99.8%), and then nucleating and growing to eventually form a monolayer of graphene. To transfer the graphene to the silicon substrate, it was coated with poly methyl methacrylate (PMMA) and cured at 150 °C for 2 h, and then transferred using a bubbling method to the silicon-based window (photosensitive area size 1 cm × 1 cm), followed by the removal of the PMMA layer with acetone to complete the construction of the graphene photodetector. As a reference, we also prepared Gr/Si devices without the HfO_X_ interfacial layer.

Finally, the physical and electrical properties of materials and devices were tested using X-ray photoelectron spectroscopy (ThermoStific, Waltham, MA, USA, K-Alpha), atomic force microscopy (Bruker, Billerica, MA, USA, Dimension Edge), Raman spectroscopy (HORIBA, Kyoto, Japan, HR-800), spectroscopic ellipsometer (VASE, M-2000D Spectroscopic Ellipsometer, J. A. Woollam, Shanghai, China), and customized photovoltaic testing system (mainly consist of Keithley 2600 source meter, probe station and laser source spectrum ranging from 400 to 650 nm).

## 3. Results and Discussion

### 3.1. Characterization of Materials and Devices

**Figure 2:**
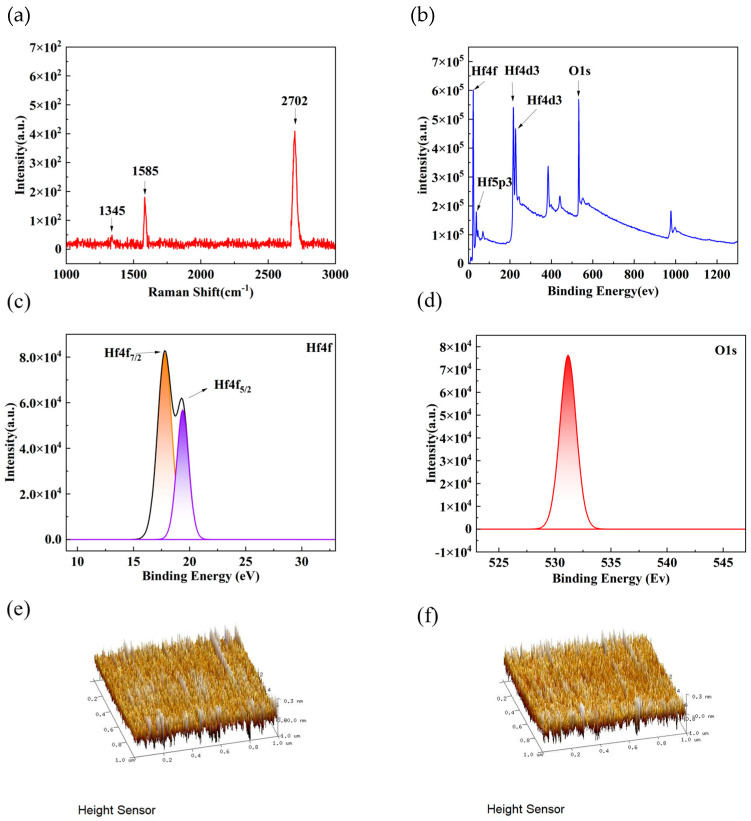
(**a**) Raman spectrum of graphene prepared by CVD; (**b**) XPS spectrum of HfO_x_ film; (**c**) XPS spectrum of Hf4f; (**d**) XPS spectrum of O1s; (**e**) AFM image of silicon wafer; (**f**) AFM image of prepared HfO_x_.

The performance of graphene-based silicon heterojunction photodetectors is partly dependent on the quality of the materials. Figure 2a shows the Raman spectroscopy test of graphene, with the G peak and 2D peak located at 1585 cm^−1^ and 2702 cm^−1^, respectively, and the D peak at 1345 cm^−1^. In addition, the D peak is very small, indicating that the graphene has almost no defects. Moreover, the I_2d_/I_g_ ratio is greater than 2, which indicates that the graphene used in the photodetector is high-quality monolayer graphene. The properties and characteristics of HfO_x_ thin films as an inserted interfacial layer determine the improvement in the performance of graphene-based silicon heterojunction photodetectors. X-ray photoelectron spectroscopy (XPS) was used for a surface chemical analysis of hafnium oxide films. The spectra confirm the presence of Hf and O elements, and the proportions calculated through peak area fitting are 30.13% and 65.36%, respectively. Other impurity elements are mainly C. This suggests that the sputtered hafnium oxide film deviates from the stoichiometric ratio of 1:2, indicating the presence of some defects [14,15]. From Figure 2c, it can be seen that the Hf 4f peak is fitted into two peaks, namely the Hf 4f_7/2_ peak and Hf 4f_5/2_ peak. Their binding energies were 17.82 eV and 19.40 eV, respectively. In Figure 2d, a characteristic O1s peak of around 531.20 eV is shown, indicating that the O element mainly exists in the form of compounds in the HfO_x_ film and is bonded with Hf atoms. These results indicate that the HfO_x_ thin films we prepared are consistent with the results of other researchers [16,17,18]. In addition, we also analyzed the etched silicon and the sputtered hafnium oxide surface on the etched silicon using atomic force microscopy. As shown in Figure 2e,f, the surface of the silicon sputtered with hafnium oxide remained uniformly smooth. The surface roughness before and after sputtering was 0.0743 nm and 0.0647 nm, respectively. After sputtering the HfO_x_ thin film, the surface roughness of the silicon is reduced. The reduced surface roughness is conducive to the formation of a heterojunction structure between graphene and silicon.

### 3.2. Performance of Gr/HfO_x_/Si Schottky Photodetector

**Figure 3:**
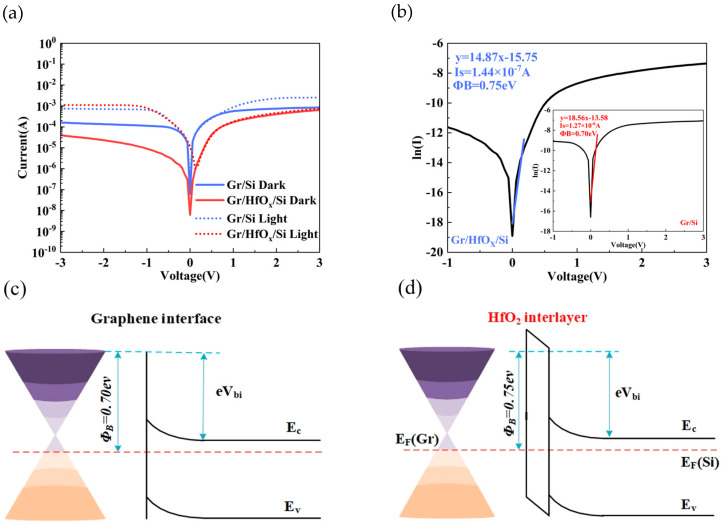
(**a**) Voltage–current curves and logarithmic curves (inset) of Gr/Si Schottky photodetector under dark conditions at room temperature; (**b**) voltage–current curves and logarithmic curves (inset) of Gr/HfO_x_/Si Schottky photodetector under dark conditions at room temperature; (**c**) linear fitting of Gr/Si Schottky junction to extract parameters; (**d**) linear fitting of Gr/HfO_x_/Si Schottky junction to extract parameters.

Figure 3a shows the current–voltage characteristics of Gr/Si and Gr/HfO_x_/Si photodetectors at room temperature (the sputtering time for HfO_x_ is 2 min, as explained below), and under dark and light conditions with an incident light intensity of 5mW/cm^2^. At a bias voltage of −2 V, the dark current of Gr/Si is 1.34 × 10^−4^ A, while the dark current of Gr/HfO_x_/Si is 2.27 × 10^−5^ A. At 0V, the dark currents for both are 6.95 × 10^−7^ A and 6.15 × 10^−9^ A, respectively, showing a significant suppression of the dark current. For the photocurrent, at a bias voltage of -2V, Gr/Si is 7.122 × 10^−4^ A, while Gr/HfO_x_/Si is 1.44 × 10^−3^ A, indicating an enhancement of the photocurrent. The insertion of the HfOx thin film reduces the reverse saturation current, achieving a low dark current. According to the theory of thermionic emission [19], the electrical transport properties of Schottky heterojunction with an inserted oxide can be expressed as follows:

(1) $$\begin{array}{ll} I & =I_{0}\left(e^{\frac{qV}{\eta kT}}-1\right)\\ & =AA^{*}T^{2}e^{-\sqrt{\chi }\delta }e^{-\frac{\Phi_{B}}{kT}}\left(e^{\frac{qV}{\eta kT}}-1\right) \end{array}$$

$I_{0}$ corresponds to the reverse saturation current, where *q*, *η*, *k*, *T*, A, $A^{*}$, and $\Phi_{B}$ represent electron charge, ideality factor, Boltzmann constant, temperature, photosensitive area, Richardson coefficient, and Schottky barrier height, respectively. The symbols χ and δ indicate the average barrier height and thickness of the inserted film. The transmittance coefficient through the interfacial layer can be expressed as $e^{-\sqrt{X}\delta }$, indicating that the interfacial layer can reduce the reverse saturation current, which is the main component of the dark current. The first part of Formula (1) can be modified as follows:

(2) $$I=I_{0}\left(e^{\frac{q\left(V-R_{S}I\right)}{\eta kT}}-1\right)$$

where *η* represents the ideality factor. *Rs* is the series resistance, and in this case, the extraction of the Schottky diode factor is calculated by the following equation:

(3) $$\frac{\partial V}{\partial (ln\;I)}=\frac{\eta kT}{q}+R_{S}I$$

Furthermore, A function *H(I)* was defined using Cheung’s [20] method.

(4) $$H(I)=V-\eta \left(\frac{kT}{q}\right)ln\left(\frac{I}{AA^{*}T^{2}}\right)={IR}_{S}+\eta \Phi_{B}$$

The above equation not only demonstrates that the inserted HfO_x_ thin film can effectively reduce the dark current but can also obtain key parameters of the Schottky diode, such as the ideality factor, reverse saturation current, and barrier height (*Φ_B_*). The typical Schottky rectifying characteristics can be observed in Figure 3b. The parameters of the Schottky diode were extracted through linear fitting. For the Gr/Si photodetector, the ideality factor, series resistance (*Rs*), and reverse saturation current are 2.29, 475 Ω, and 1.27 × 10^−6^ A, respectively. For the Gr/HfO_x_/Si photodetector, the ideality factor, series resistance (*Rs*), and reverse saturation current were 2.86, 737 Ω, and 1.44 × 10^−7^ A, respectively. Additionally, the Schottky barrier (*Φ_B_*) heights were calculated as 0.70 eV and 0.75 eV. In summary, these results indicate that the inserted hafnium oxide interfacial layer can increase the Schottky barrier height (*Φ_B_*), and suppress the reverse saturation current and dark current.

As a comparative experiment, the light response mechanism can be explained by the band theory shown in Figure 3c. When graphene comes into contact with n-Si, a Schottky junction is automatically formed, resulting in the formation of a built-in electric field (eV_bi_) and a Schottky barrier *Φ_B_* on the contact surface. However, due to the imperfections of graphene and the presence of a large number of dangling bonds on the surface-treated silicon surface, high-density surface states are present at the interface of the actual Gr/Si, which lead to the formation of a partially dark current due to carrier recombination at the surface [21,22,23]. In addition, the lower barrier height allows thermally excited electrons to tunnel through the interface, resulting in a reverse saturation current. Figure 3d shows that, after inserting the HfOx interface layer, graphene separates from the silicon surface and the barrier height (*Φ_B_*) increases, preventing the thermal generation of carriers across the barrier, and thus inhibiting the generation of a reverse saturation current [24]. The presence of fewer structural defects in the uniform interfacial layer helps to reduce surface state density and decrease the recombination current. However, in some cases, moderate defects can provide trap levels and improve the efficiency of carrier transport, which has a positive impact on the performance of the photodetector. However, due to the non-stoichiometric ratio of the sputtered HfO_x_ interfacial layer, a certain number of defects will be generated in the interfacial layer, providing transition channels for carriers. When this structure is illuminated, the photo-generated carriers are separated under the action of the built-in electric field, with holes being injected into the valence band of graphene and electrons moving into silicon [22]. The HfO_x_ interfacial layer provides a transport channel for holes, resulting in a larger photocurrent. In summary, the higher Schottky barrier, lower surface state, and unsaturated oxygen vacancies generated by the HfO_x_ interfacial layer help to reduce the dark current and enhance carrier transport.

**Figure 4:**
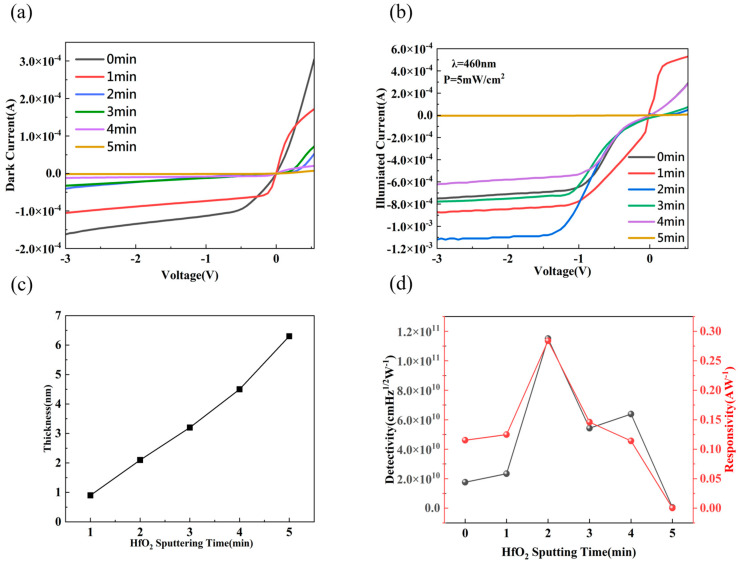
Current–voltage characteristics of graphene/Si Schottky photodetectors with different HfO_x_ interface layers: (**a**) under dark conditions; (**b**) under light illumination with an incident power of 5 mW/cm^2^ and a wavelength of 460nm; (**c**) variation in film thickness with sputtering time; (**d**) responsivity and detectivity.

As mentioned above, HfO_x_ plays a crucial role in the Gr/Si Schottky junction, contributing to an improvement in the photodetector’s performance. Figure 4a shows the graphene/Si heterojunction photodetectors with different sputtering times on the hafnium oxide interfacial layer. These photodetectors exhibit obvious rectifying characteristics, both with and without HfO_x_. When biased at −2 V, the dark current of the photodetector with a sputtering time of 2 min decreases by approximately six times compared to the one without a sputtered HfO_x_ interfacial layer, reduced from 1.34 × 10^−4^ A to 2.27 × 10^−5^ A. With the increase in the sputtering time of the HfO_x_ interfacial layer, the dark current further decreases to 1.49 × 10^−6^ A, reducing by two orders of magnitude. The increase in sputtering time leads to an increase in the thickness of the interfacial layer, thereby exerting a greater suppression effect on the dark current.

Figure 4b shows the photocurrent curves under the conditions of an incident light wavelength of 460 nm and an incident light intensity of 5 mw/cm^2^ with different thicknesses of the HfO_x_ interfacial layer. When there is no HfO_x_ interfacial layer, the photocurrent saturates under a bias of −1 V, while under reverse bias, the increase in photocurrent is mainly due to the effect of minority carriers. When the sputtering time is 1 min, 2 min, and 3 min, the photocurrent increases; when the sputtering time is 2 min, the current increases from 7.10 × 10^−4^ A to 1.44 × 10^−3^ A. Introducing the hafnium oxide interfacial layer can improve the band structure between graphene and silicon and reduce the influence of surface states. By adjusting the band structure, the electron transmission and injection efficiency between graphene and silicon can be enhanced, reducing the reflectance and scattering of photoelectrons. This leads to the capture and transfer of more photoelectrons to the electrode, thereby increasing the generation of a photocurrent [25]. With the increase in sputtering time, the thickness of the HfO_x_ interfacial layer increases, and the tunneling of carriers is suppressed, resulting in a rapid decrease in photocurrent.

Figure 4c shows the thickness of HfO_x_ thin films measured using an Ellipsometer (SE) at different sputtering times. The thickness of the HfO_x_ thin film increases almost linearly with the increase in sputtering time. In order to quantitatively evaluate the performance of the photodetector, the responsivity and detectivity are calculated using the following equations:

(5) $$R=\frac{I_{ph}}{P_{in}},D^{*}=\frac{A^{1/2}R}{\sqrt{2eI_{d}}}$$

Among them, *I_ph_* is the photocurrent, *P_in_* is the incident light power, *I_d_* is the dark current, and *A* is the device area. From Figure 4d, it can be seen that the device performs best at a sputtering time of 2 min, exhibiting a performance of approximately 0.284 A/W and 1.15 × 10^11^ cmHz^1/2^W^−1^ at a wavelength of 460 nm. This is because the increase in the thickness of the HfO_x_ interfacial layer gradually reduces the dark current, and the interfacial layer causes the photocurrent to initially increase and then decrease. Therefore, the device with an interfacial layer thickness of about 2 nm exhibits the optimal performance, corresponding to a sputtering time of 2 min for the photodetector. The performance of this Gr/HfO_x_/Si photodetector is comparable to that of other similar photodetectors [26,27,28].

**Figure 5:**
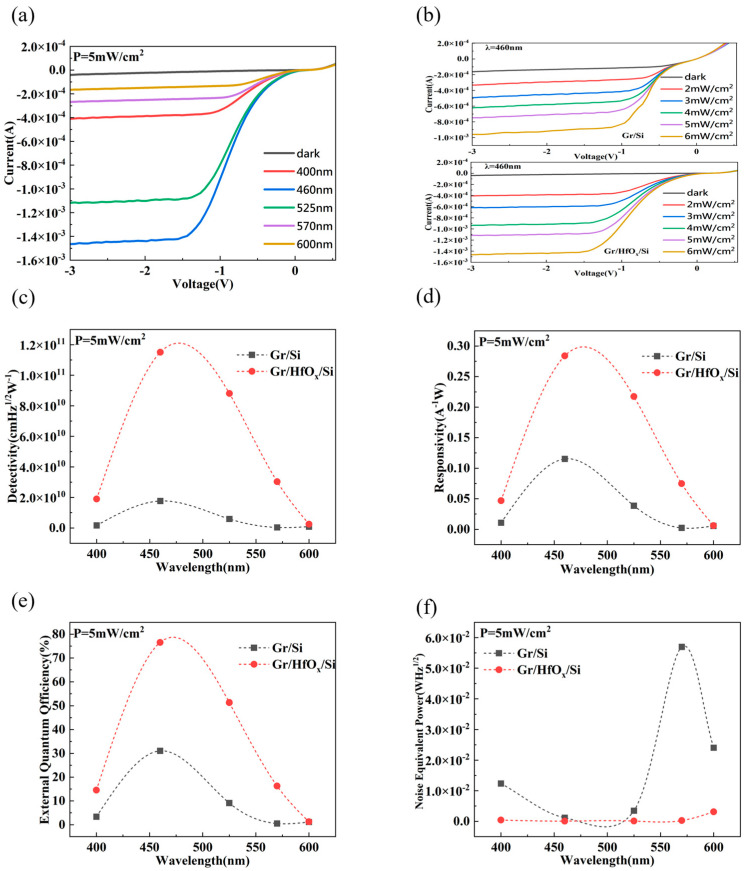
(**a**) I–V curves of Gr/HfO_x_/Si Schottky photodetectors at different incident light wavelengths; (**b**) I–V curves of Gr/HfO_x_/Si Schottky photodetectors at different light powers; (**c**) detectivity of Gr/HfO_x_/Si Schottky photodetectors and Gr/Si Schottky photodetectors at different incident light wavelengths; (**d**) responsivity; (**e**) quantum efficiency; (**f**) noise equivalent power.

Figure 5a shows the current–voltage characteristics curve of the Gr/HfO_x_/Si photodetector under the same light intensity. The photodetector responds in the range of 400 nm–600 nm, with the best response at 460 nm. To characterize the current–voltage characteristics of the Gr/HfO_x_/Si photodetector and the Gr/Si photodetector at different powers, we chose an incident light of 460 nm, as shown in Figure 5b. With the increase in irradiance density, the current of both Gr/HfO_x_/Si and Gr/Si photodetectors shows a monotonic increasing trend. Generally, when the light intensity increases slightly, from darkness to 6 mW/cm^2^, an increase in photocurrent can be observed, indicating that both types of photodetectors have good low-light detection capabilities. The experimental results demonstrate that the light response of the two photodetectors exhibits nearly linear changes, with the photocurrent gradually increasing as the light intensity rises, suggesting that the introduction of the HfO_x_ thin film has little significant impact on the linearity of the light power response. To further study the performance of the photodetector with and without the HfO_x_ interfacial layer, key parameters of the photodetector, such as responsivity, detectivity, external quantum efficiency, and noise equivalent power, are quantitatively analyzed. Figure 5c,d show a relative comparison between the graphene/silicon photodetector with a 2 min sputtered HfO_x_ interfacial layer and the graphene/silicon photodetector without a interfacial layer under a constant power of 5 mW/cm^2^. Both responsivity and detectivity show a significant improvement. At a wavelength range of 400–600 nm, the photodetector exhibits peak sensitivity at 460 nm, with responsivity increasing from 0.115 A/W to 0.284 A/W, and detectivity increasing from 1.76 × 10^10^ cmHz^1/2^W^−1^ to 1.15 × 10^11^ cmHz^1/2^W^−1^. The photodetector with the HfO_x_ interfacial layer shows higher responsivity and detectivity compared to the photodetector without an interfacial layer. The external quantum efficiency and noise equivalent power are calculated using the following formulas:

(6) $$EQE=\frac{I/e}{P/hv}\;\;NEP=\frac{A^{1/2}}{D^{*}}$$

In the equation, *I/e* represents the number of electron–hole pairs generated per unit time. *P/hv* represents the number of photons incident per unit time, *A* represents the area of the photodetector, and $D^{*}$ represents the detectivity of the photodetector.

Figure 5e,f show the quantum efficiency and noise power of the Gr/HfO_x_/Si Schottky photodetector and the Gr/Si Schottky photodetector at different wavelengths at room temperature and with an incident power of 5 mW/cm^2^. At a wavelength of 460 nm, the quantum efficiency of the Gr/HfO_x_/Si Schottky photodetector reaches 76.5%, more than double compared to the 31.1% of the Gr/Si photodetector. This provides new ideas and solutions to improve the conversion efficiency of graphene-based silicon solar cells. Additionally, the addition of the interfacial layer reduced the noise equivalent power by an order of magnitude. The noise equivalent power at a wavelength of 460 nm decreased from 1.16 × 10^−3^ WHz^1/2^ to 8.75 × 10^−5^ WHz^1/2^, demonstrating the working capability of this photodetector under low signal conditions. A lower noise equivalent power implies a higher signal-to-noise ratio, lower minimum detectable signal, and a larger dynamic range for the detector.

**Figure 6:**
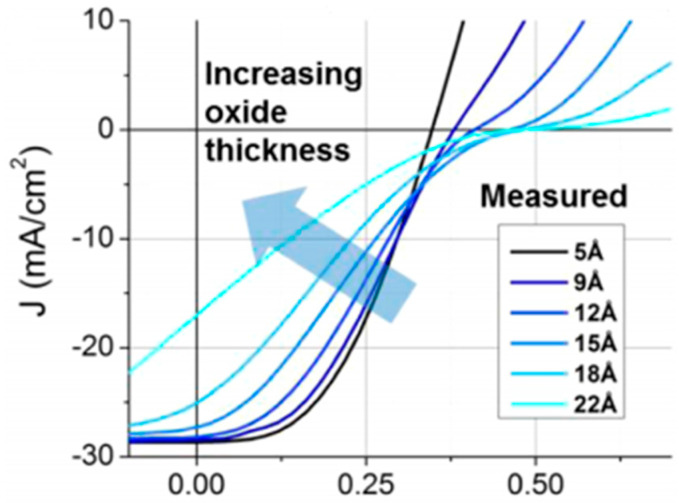
I–V characteristics of graphene/n-silicon devices with different oxide layer thicknesses [11].

In addition, Song et al. [11] found that with the increase in interface oxide thickness, the photocurrent of the graphene-based silicon heterojunction decreases and the light response decreases at low bias voltages, as shown in Figure 6.

**Figure 7:**
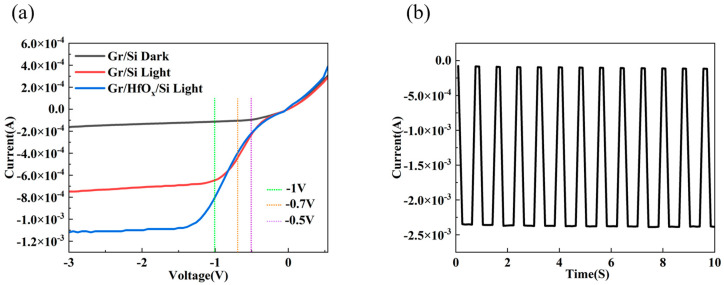
(**a**) I–V characteristics of Gr/HfO_x_/Si and Gr/Si photodetectors under 460 nm wavelength light intensity of 5 mW/cm^2^; (**b**) time-dependent photoresponse of wavelength −1 V and 650 nm light illumination (10 mW/cm^2^).

Our research also indicates that the HfO_x_ interfacial layer grown by magnetron sputtering has little effect on the low-voltage response of the device, as shown in Figure 7a. It can be clearly seen that the Gr/HfO_x_/Si photodetector only experiences a slight suppression of the photocurrent within a range of about 0.7V. At a bias voltage of 0.7 V, the photocurrent without the interfacial layer is 4.46 × 10^−4^ A, slightly higher than the 3.97 × 10^−4^ A for the device with the HfO_x_ interfacial layer. At a bias voltage of −1 V, the photodetector with the HfO_x_ interfacial layer has a photocurrent of 8.10 × 10^−4^ A, which is higher than the 6.41 × 10^−4^ A for the graphene/silicon photodetector without an interfacial layer, demonstrating the excellent detection performance of the Gr/HfO_x_/Si photodetector at low bias voltages. Figure 7b shows the I–T curve of the Gr/HfO_x_/Si photodetector at an incident light wavelength of 650 nm and an intensity of 10 mW/cm^2^. The I_light_/I_dark_ ratio is 3.1 × 10^1^, indicating a good on/off ratio.

The Gr/HfO_x_/Si photodetector demonstrates an exceptional performance, with a noise equivalent power of 8.75 × 10^−5^ pW/Hz^1/2^, a zero bias dark current of 6.15 nA, and a quantum efficiency of 76.5%. A comparison with prior research, highlighted in Table 1, reaffirms its superior capabilities. Nevertheless, enhancements are warranted in responsivity and detection sensitivity, suggesting further scope for improvement in these areas, underscoring the excellent performance delivered by the Gr/HfO_x_/Si photodetector.

## 4. Conclusions

This study demonstrates that magnetron sputtered non-stoichiometric HfO_x_ film can serve as a dielectric layer for graphene/silicon Schottky junction photodetectors. The current–voltage (IV) characteristics show that the reverse saturation current is suppressed and the dark current of the photodetector decreases significantly due to the insertion of the HfO_x_ film. The key factors extracted from the ideal equation of thermionic emission theory indicate that the barrier height is significantly enhanced by inserting the HfO_x_ film. Additionally, the photocurrent increased by 2.03 times due to the introduction of the conductive channel provided by the HfO_x_ film. Under −2 V reverse bias, the responsivity reaches 0.284 A/W at 460 nm illumination. The detectivity and noise equivalent power are improved by the introduction of the HfO_x_ interfacial layer, achieving 1.15 × 10^11^ Jones and 8.75 × 10^−5^ pW/Hz^1/2^, respectively. The IV characteristics show that the photocurrent is proportional to the incident optical power and remains stable with increasing bias voltage. Furthermore, the detection capability of the graphene/silicon photodetector at low bias voltages is well maintained after the insertion of the HfO_x_ interfacial layer. This work demonstrates that the HfO_x_ interfacial layer has a dual function, suppressing the dark current and enhancing the photocurrent, providing a new approach to improve the performance of photodetectors.

## Figures and Tables

**Table 1:** Key photodetection performance of Gr/Si photodetectors.

Device Structure	λ nm	Responsivity A/W	D* Jones	NEP pW/Hz^1/2^	I_dark_ nA	EQE(%)	Ref.
Gr/HfO_x_/Si	460	0.284	1.15 × 10^11^	8.75 × 10^−5^	6.15	76.5	this work
Gr/Si	890	0.73	4.2 × 10^12^	0.075	9.3	65.0	[29]
Gr/Si	730	0.435	2.1 × 10^8^	33			[30]
Gr/Al_2_O_3_/Si	658	0.75	3.1 × 10^12^				[10]
Gr/H-Gr/Si	532	0.245	2.3 × 10^11^		100		[31]
Gr/h-BN/Si	725		2.83 × 10^10^				[32]
P3HT–Gr/Si	540	0.78	2.6 × 10^10^	0.14	40		[28]

## Data Availability

No new data were created or analyzed in this study. Data sharing is not applicable to this article.
